# Causal machine learning uncovers conditions for convective intensification driven by organic and sulfate aerosols

**DOI:** 10.1038/s41598-025-28939-x

**Published:** 2025-12-29

**Authors:** Dié Wang, Jie Xi Li, Jun Lu

**Affiliations:** 1 Environmental Science and Technologies Department, Brookhaven National Laboratory, 98 Rochester St, Upton, 11937 NY USA; 2 Institute for Atmospheric and Climate Science, ETH Zurich, Universitätstrasse 16, Zurich, 8092 Switzerland; 3 Applied Mathematics and Statistics, Stony Brook University, 100 Nicolls Road, Stony Brook, 11794 NY USA; 4 School of Public Health, University of Illinois Chicago, 1200 West Harrison Street, Chicago, 60607 IL USA

**Keywords:** Aerosol-cloud interaction, Deep convective cloud, Causal inference, Causal discovery, Machine learning, Climate sciences, Environmental sciences, Natural hazards

## Abstract

Aerosols are often hypothesized to invigorate deep convective clouds (DCCs), but observational evidence remains limited and inconclusive. Clarifying this hypothesis is critical for regions vulnerable to thunderstorms and flooding, particularly highly polluted coastal cities. Leveraging a novel causal discovery–inference pipeline and high-resolution observations near Houston, TX, we identify multiple causal pathways among aerosols (mostly organic and sulfate), DCCs, and meteorological factors. However, a direct causal link from aerosols to DCCs is found to be uncommon, occurring in less than 35% of analyzed scenarios, and is characterized by strong conditionality and nonlinearity. When aerosol impacts on DCCs do occur, they can be substantial, enhancing DCC core heights by approximately 1.7 km, with 92% of this effect concentrated in warmer-phase cloud regions. Notably, the presence of sea breezes and the inclusion of all measured aerosol particles each enhance DCCs in over 95% of aerosol-sensitive cases.

## Introduction

Aerosols–tiny solid or liquid particles suspended in the atmosphere–play a fundamental role in cloud microphysics by acting as cloud condensation nuclei (CCN), around which water vapor condenses to form cloud droplets. Elevated aerosol concentrations have been shown to modulate the intensity of deep convective clouds (DCCs), such as thunderstorms, potentially altering their rainfall distribution, duration, and lightning intensity^1–3^. However, the magnitude, sign, and meteorological dependence of aerosol impacts on DCCs remain active areas of research and debate within the atmospheric science community^4^. Clarifying these effects is particularly important for coastal metropolitan regions, where extreme weather events are frequent and aerosol burdens are elevated due to emissions from industrial facilities, urban infrastructure, and maritime activity^5^. In cities such as Houston, TX, which are highly susceptible to heavy rainfall and flooding, including from tropical cyclones, the potential for aerosol-induced enhancement of convective intensity poses both scientific and societal challenges, with direct implications for urban planning, disaster risk assessment, and climate adaptation strategies^5^.

Two dominant perspectives exist in aerosol-DCC interactions. On one side, some studies suggest that more aerosols invigorate DCCs through cloud microphysics-dynamics interactions^3,6–8^. For example, in the “cold-phase invigoration” theory, high aerosol concentrations lead to more but smaller cloud droplets. This slows down raindrop formation, allowing more liquid water to rise above the freezing level. When that water freezes, it releases extra latent heat, making the updrafts stronger and the DCCs more intense^9–11^. On the other side, other studies argue that increased aerosol loading is more likely to suppress DCCs, particularly when realistic entrainment and hydrometeor loading effects are accounted for^12–14^. Some also suggest that aerosol impacts may be negligible, especially when compared to the large natural variability within DCC populations^15^. These results hint at the fact that all of these scenarios may be plausible, as they were studied independently under different conditions favorable for DCC development and using distinct methodological approaches. To resolve this long-standing debate, sufficient and conclusive observational evidence is essential, but progress is impeded by three key challenges:

First, measuring critical properties of DCCs remains technically demanding. For instance, direct measurements of updraft velocity within convective cores, the central variable in evaluating the aerosol invigoration hypotheses, are extremely limited, constrained to a few specific locations and/or short periods^16,17^. Furthermore, in-cloud supersaturation, a critical factor in enabling warm-phase aerosol invigoration through enhanced latent heat release from additional aerosol activation, cannot be measured directly at this stage and needs to be informed using indirect retrievals, introducing uncertainties into the conclusions^18,19^. Moreover, entrainment profiles are notoriously difficult to quantify with precision^20^. It limits our ability to test humidity-entrainment invigoration hypothesis^21^, where aerosol-rich DCCs may detrain more condensate, increasing ambient humidity and promoting conditions that support large-scale ascent and stronger convection. Overall, the scarcity of relevant measurements significantly prevents us from rigorously evaluating these mechanisms.

Second, uncovering causal links between aerosols and DCCs based solely on observational data continues to pose a major methodological obstacle^4^. This is, in fact, rooted in the atmospheric science community’s longstanding reliance on correlation-based analyses, which often fall short of attributing causal directions among variables. At its core, this reveals a persistent, but often overlooked gap in observational data analysis: the absence of causal reasoning. Addressing central question of this study-*whether aerosol loading is a contributing cause of variability in DCC intensity*–demands a fundamental shift toward analytical frameworks that explicitly resolve causality. Despite growing recognition of its importance^22,23^, the application of causal discovery remains limited and far from routine practice in the atmospheric science community.

Third, precisely quantifying the magnitude and significance of aerosol effects on DCCs represents a persistent difficulty. This arises from the inherent impossibility of observing the same convective system in nature under both clean and polluted conditions while holding all other factors unchanged. In essence, we are attempting to answer a counterfactual question: *Would the DCC have been stronger if aerosol concentrations had been higher?* This question may seem addressable only through numerical simulations by varying relevant model parameters^15,24–26^. However, promisingly, if the aerosol–DCC relationship can be properly deconfounded by removing the effects of meteorological variables, estimating the true causal effects would no longer be out of reach. Previous observational studies have made significant progress along these lines, mostly through regression models^2,7,27,28^. For example, higher aerosol number concentrations have been linked to shallower DCCs in Argentina^28^ and to narrower convective cells in Houston, with the latter relationship modulated by the synoptic environment^29^. However, these models alone are generally recognized as insufficient for drawing reliable causal inference^30,31^, highlighting the need for causal thinking and advanced statistical frameworks to address or mitigate these limitations^32^.

In this study, we apply a causal analysis framework to a comprehensive observational dataset with the goal of providing evidence for or against aerosol impacts on DCCs in a coastal location (Houston, TX). The observations used here were collected from June to September, 2022, as part of the Department of Energy’s Atmospheric Radiation Measurement (ARM) TRacking Aerosol Convection Interactions ExpeRiment (TRACER^5,33^), and summarized in^34^. Our first step integrates causal discovery algorithms to infer directional relationships among aerosol loading, meteorological factors, and DCC properties. This step also allows for identifying a minimal set of covariates required for unbiased inference–a key advancement over heuristic or ad hoc variable selection. We then apply double/debiased machine learning (DML^35^), a state-of-the-art causal inference method that accommodates complex causal relationship and mitigates confounding bias, to quantify the average effect of aerosols on DCCs after adjusting for covariates. Together, this study represents one of the first applications of a full causal discovery–inference pipeline to the field of aerosol–cloud interaction, offering a statistically rigorous and generalizable framework for isolating and estimating causal effects solely from observational datasets. Note that this study is a follow-up or extension of^34^ based on the same dataset but employing different methods.

The rest of this paper is organized as follows. The Results section presents the main causal relationships identified among key observables using causal discovery models, with a particular focus on the link between aerosol number concentration and the height of the DCC core. It also quantifies the effects of aerosols on DCC height under different scenarios using causal inference models. The Discussion section summarizes the main conclusions of the study and highlights key challenges and uncertainties associated with this and related work. Finally, the Methodology section describes the datasets and causal modeling approaches used in this study (also summarized in Figure S3).

## Results

### Aerosols and DCCs are causally linked

**Fig. 1:**
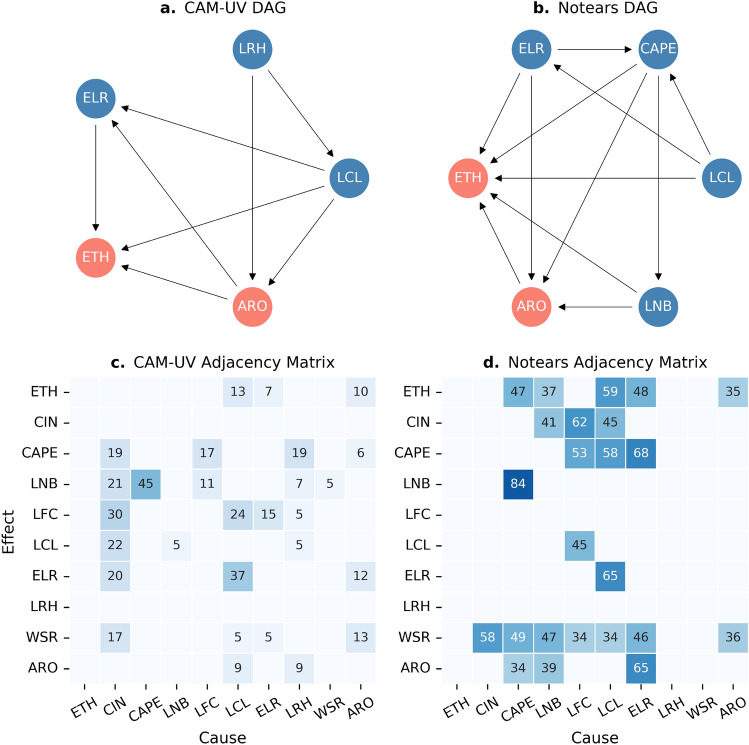
DAGs inferred using CAM-UV (**a**) and NOTEARS (**b**) are shown, displaying only the causal links directly connected to ARO and ETH or those lying along their causal pathways for visual clarity. An arrow from variable *x* to *y* implies that changes in *x* are inferred to causally affect *y*. The corresponding adjacency matrices, indicating frequency of each direction from bootstrap iterations (100 times) for 576 sensitivity tests (57,600 model runs in total), are shown in (**c**) for CAM-UV and (**d**) for NOTEARS. Only the top 23 links are illustrated, representing the most robust causal directions. The sensitivity tests are listed in Table S2 in the supplemental material. The aerosol properties (ARO) include total aerosol number concentrations ($N_{cn}$), total concentrations including ultrafine particles ($N_{ufp}$), and CCN number concentrations at supersaturation levels ranging from 0.1% to 1% (used individually in the models). WSR = low-level wind shear between the surface and 5 km; LFC = level of free convection; CIN = convective inhibition.

In Fig. 1a and b, we present the inferred causal structure among DCC 30-dBZ/15-dBZ radar echo top height (ETH), aerosol number concentrations (ARO), and key meteorological variables, derived from two nonlinear causal discovery models. Here, the 30-dBZ or 15-dBZ ETH represents the maximum value reached by isolated DCCs during their lifecycles and serves as a proxy for updraft or DCC intensity^28,36^. Figure 1a and b are also known as Directed Acyclic Graphs (DAG,^37^). A DAG is a graphical representation of causal relationships in which nodes represent observed variables and directed edges (arrows) indicate the direction of causality, pointing from cause to effect.

Figure 1c and d show the corresponding adjacency matrices, which summarize the probability of each inferred causal direction based on 100 bootstrapped iterations for each of the 576 sensitivity tests, a total of 57,600 model runs (see Table S1 for details on sensitivity tests). A lower percentage in the adjacency matrices does not necessarily indicate an incorrect causal direction; instead, it may reflect a combination of high nonlinearity and limited robustness under certain conditions^22^.

The two causal discovery models used are the Causal Additive Model with Unobserved Variables (CAM-UV;^38^) and the nonlinear Non-combinatorial Optimization via Trace Exponential and Augmented Lagrangian for Structure Learning (NOTEARS;^39^). This combination is a core part of our approach, as the two models differ in their assumptions, sensitivity to algorithm and parameter choices, and ability to handle unmeasured confounders (see Section 4.2). Here, a confounder is a variable that influences both the exposure (e.g., ARO) and the outcome (e.g., ETH), thereby inducing a spurious association between them (see Fig. S4 for more information). As a result, they may learn distinct causal graphs from the same dataset, despite the inherent difficulty of uncovering relationships in such complex systems, which are often governed by strong feedbacks and are highly sensitive to multi-scale regimes. If both results are plausible, this could indicate the presence of multiple causal pathways favored under different model assumptions. If only one result is plausible, it still provides valuable insights into each model’s strengths and limitations for revealing causal relationships in atmospheric science. Discrepancies in the identified causal links may also highlight areas that warrant further investigation using additional models, alternative approaches, or independent datasets, thereby guiding future research directions.

As shown in Fig. 1a and b, interestingly, two model results do share some structural similarities, although notable discrepancies remain. Both models reveal a “direct” causal link from ARO to ETH, indicating that changes in aerosol loading alter DCC intensity. However, the bootstrap probability for the ARO $\rightarrow$ ETH link remains relatively low (Fig. 1c and d), suggesting a highly complex and nonlinear relationship between aerosols and DCCs. In fact, this “direct” causal pathway likely masks a more intricate set of interactions involving cloud microphysics and dynamical processes^24^ that are not captured here due to the absence of in-cloud updraft velocity and direct microphysical measurements during TRACER (some of them could be confounders). Moreover, it does not rule out the possibility that aerosols affect ETH through microphysical pathways alone, without exerting a significant influence on updraft intensity^40^. Note that the presence of these unmeasured/undiscovered confounders in causal discovery models may result in the appearance of a direct link in DAGs, even when only an indirect pathway exists in reality^22^.

Despite this direct link, several meteorological variables, including the environmental lapse rate (ELR), lifting condensation level (LCL), convective available potential energy (CAPE), and level of neutral buoyancy (LNB), exhibit causal connections to both ETH and ARO. Overall, CAM-UV identify fewer of these variables (Fig. 1a), revealing a simpler causal structure compared to NOTEARS (Fig. 1b). This could be due to the removal of edges involving unmeasured or undiscovered confounders in CAM-UV, which are not identified using NOTEARS (see Section 4.2).

More importantly, the causal pathways through which ARO influences ETH differ across models. In particular, CAM-UV suggests that the effect of ARO on ETH is mediated by ELR via the pathway ARO $\rightarrow$ ELR $\rightarrow$ ETH, highlighting ELR’s role as a mediator. A mediator is a variable that lies on the causal pathway between two variables (see Fig. S4). In contrast, NOTEARS indicates only arrows directed toward both ETH and ARO from ELR, identifying it as a confounder. The role of ELR as either a mediator or a confounder can be physically well justified: ELR reflects atmospheric instability, which could affect convective updraft strength and, in turn, the height of the convective core (ELR $\rightarrow$ ETH, 65%). At the same time, it could also influence vertical mixing and boundary layer depth, thereby modulating ARO distributions within the lower troposphere (ELR $\rightarrow$ ARO)^41^. The reverse direction (ARO $\rightarrow$ ELR) is not implausible, given that elevated aerosol number concentration can modify vertical temperature profiles and sensible heat fluxes through radiative effects, impacting atmospheric instability^42^. However, this causal direction appears less systematic, as indicated by the low bootstrap probability (12%) in CAM-UV (Fig. 1c)

Based on CAM-UV, LCL is also an intuitive confounder (Fig. 1a). A lower LCL implies that air parcels do not need to rise high before condensation occurs, releasing latent heat earlier and modifying DCC vertical structure, ultimately impacting ETH. The LCL $\rightarrow$ ARO pathway is plausible because a lower LCL often corresponds to a shallower boundary layer, which may confine aerosols to lower altitudes, altering aerosol concentrations near the surface. Similarly, LNB is well known to influence the height of the convective core, along with entrainment and buoyancy dilution^43^ (see results from NOTEARS, Fig. 1b). It can also affect aerosol distribution by controlling how far lifted air masses (and the aerosols they contain) are transported vertically in the troposphere.

**Table 1:** Identified backdoor paths based on learned DAG from CAM-UV (Fig. 1a) and NOTEARS (Fig. 1b) and the variables that should be controlled for to block each path (deconfounding ARO $\rightarrow$ ETH) when estimating ARO effects on ETH. Variable abbreviations are as follows: ELR = environmental lapse rate, LRH = mean relative humidity below 5 km, LCL = lifting condensation level, CAPE = convective available potential energy, and LNB = level of neutral buoyancy.

Path No.	Backdoor paths	Control variables to block the path
CAM-UV		
1	ARO $\leftarrow$ LCL $\rightarrow$ ETH	LCL
2	ARO $\leftarrow$ LCL $\rightarrow$ ELR $\rightarrow$ ETH	LCL or ELR
3	ARO $\leftarrow$ LRH $\rightarrow$ LCL $\rightarrow$ ETH	LCL or LRH
4	ARO $\leftarrow$ LRH $\rightarrow$ LCL $\rightarrow$ ELR $\rightarrow$ ETH	LCL or LRH or ELR
NOTEARS		
5	ARO $\leftarrow$ ELR $\rightarrow$ ETH	ELR
6	ARO $\leftarrow$ ELR $\leftarrow$ LCL $\rightarrow$ ETH	ELR or LCL
7	ARO $\leftarrow$ ELR $\leftarrow$ LCL $\rightarrow$ CAPE $\rightarrow$ ETH	ELR or LCL or CAPE
8	ARO $\leftarrow$ ELR $\leftarrow$ LCL $\rightarrow$ CAPE $\rightarrow$ LNB $\rightarrow$ ETH	ELR or LCL or CAPE or LNB
9	ARO $\leftarrow$ ELR $\rightarrow$ CAPE $\rightarrow$ ETH	ELR or CAPE
10	ARO $\leftarrow$ ELR $\rightarrow$ CAPE $\leftarrow$ LCL $\rightarrow$ ETH	N/A or (LCL & CAPE) or (CAPE & ELR)
11	ARO $\leftarrow$ ELR $\rightarrow$ CAPE $\rightarrow$ LNB $\rightarrow$ ETH	ELR or CAPE or LNB
12	ARO $\leftarrow$ CAPE $\rightarrow$ ETH	CAPE
13	ARO $\leftarrow$ CAPE $\leftarrow$ ELR $\rightarrow$ ETH	CAPE or ELR
14	ARO $\leftarrow$ CAPE $\leftarrow$ LCL $\rightarrow$ ETH	CAPE or LCL
15	ARO $\leftarrow$ CAPE $\rightarrow$ LNB $\rightarrow$ ETH	CAPE or LNB
16	ARO $\leftarrow$ CAPE $\leftarrow$ ELR $\leftarrow$ LCL $\rightarrow$ ETH	CAPE or ELR or LCL
17	ARO $\leftarrow$ CAPE $\leftarrow$ LCL $\rightarrow$ ELR $\rightarrow$ ETH	CAPE or ELR or LCL
18	ARO $\leftarrow$ LNB $\rightarrow$ ETH	LNB
19	ARO $\leftarrow$ LNB $\leftarrow$ CAPE $\rightarrow$ ETH	LNB or CAPE
20	ARO $\leftarrow$ LNB $\leftarrow$ CAPE $\leftarrow$ ELR $\rightarrow$ ETH	LNB or CAPE or ELR
21	ARO $\leftarrow$ LNB $\leftarrow$ CAPE $\leftarrow$ LCL $\rightarrow$ ETH	LNB or CAPE or LCL
22	ARO $\leftarrow$ LNB $\leftarrow$ CAPE $\leftarrow$ LCL $\rightarrow$ ELR $\rightarrow$ ETH	LNB or CAPE or LCL or ELR
23	ARO $\leftarrow$ LNB $\leftarrow$ CAPE $\leftarrow$ ELR $\leftarrow$ LCL $\rightarrow$ ETH	LNB or CAPE or LCL or ELR

Interestingly, CAPE exhibits no direct or indirect causal links to ETH or ARO in the CAM-UV DAG, whereas in the NOTEARS DAG, it plays a multifaceted role with its influence varying across different causal pathways. In particular, CAPE acts as a confounder in the ARO $\rightarrow$ ETH relationship by directly influencing both ETH and ARO (Path 12 in Table 1), and also indirectly affecting ARO through LNB (Path 15 in Table 1). This structure aligns with physical intuition: CAPE quantifies the potential energy available to support air parcel ascent and strongly regulates convective updraft strength. Thus, CAPE serves as a key determinant of both the level a parcel is expected to reach (LNB) and the height it ultimately achieves (ETH)^44^. Note that bootstrapping analysis reveals a substantially lower probability for the CAPE $\rightarrow$ ETH link (47%) compared to the CAPE $\rightarrow$ LNB link (84%) (Fig. 1d). This contrast likely reflects the greater complexity and nonlinearity of the CAPE–ETH relationship, which is largely influenced by entrainment and mixing processes that reduce parcel buoyancy and constrain the vertical extent of DCCs^43^. In addition, as an indicator of atmospheric instability, CAPE can shape aerosol distribution by regulating the strength of vertical mixing and energy transport within the boundary layer and beyond.

CAPE also functions as a mediator in several causal pathways (i.e., Paths 9, 11, 13, 14, 16, and 17 in Table 1), where it transmits part of the influence of LCL or ELR onto ARO or ETH. The roles of LCL and ELR in modulating CAPE are relatively robust, with bootstrap probabilities exceeding 58%, consistent with their shared reflection of lower-tropospheric instability. For example, ELR impacts CAPE, as a steeper ELR enhances atmospheric instability, thereby contributing to the accumulation of positive buoyancy. In other scenarios, CAPE serves as a collider (a variable that is jointly influenced by two or more other variables, see Fig. S4), such as in the pathway ARO $\leftarrow$ ELR $\rightarrow$ CAPE $\leftarrow$ LCL $\rightarrow$ ETH (Path 10 in Table 1), where it is influenced by two or more upstream variables. These findings underscore not only the central role of CAPE but also the intricate, context-dependent nature of aerosol-DCC interaction processes^45^, in which individual variables often play multiple roles within the causal network.

Overall, this section reveals multiple plausible causal pathways from ARO to ETH, both *direct* and *indirect* through aerosol-induced modifications of meteorological conditions. This directly addresses our first research question and support the conclusion that aerosol loading is a contributing cause of variability in DCC intensity based on observational evidence. However, such causal link is identified infrequently (10% for CAM-UV, 35% for NOTEARS), highlighting its conditional and episodic nature.

### Isolating aerosol effects via backdoor criterion

Having established the DAG and identified a potential causal link between ARO and ETH, we next verify this connection and quantify the effect of ARO on ETH. A central challenge lies in isolating the ARO effect by adequately controlling for confounding influences. To address this, we apply the backdoor criterion^31^ to identify a minimal sufficient set of covariates for adjustment. This principle is a cornerstone of modern causal inference and ensures unbiased estimation by avoiding over- or under-adjustment^30^.

More specifically, given an exposure *D* (e.g., ARO) and an outcome *Y* (e.g., ETH), a set of variables *X* satisfies the backdoor criterion if: (1) No variable in *X* is a descendant of *D*, and (2) *X* blocks all backdoor paths from *D* to *Y*, that is, all paths that begin with an arrow pointing into *D*. In simpler terms, we must adjust for variables that confound both *D* and *Y*, but not for variables that lie on the causal pathway from *D* to *Y* (i.e., mediators), or that are common effects of both *D* and *Y* (i.e., colliders).

Based on the causal graphs or DAGs revealed by CAM-UV (Fig. 1a) and NOTEARS (Fig. 1b), we identify 4 and 19 backdoor paths from ARO to ETH, respectively, as detailed in Table 1. To block all of these paths, we must adjust for at least one key variable (LCL) for CAM-UV and three key variables (ELR, CAPE, and LNB) for NOTEARS. Details on how each individual backdoor path can be blocked are provided in Table 1.

Essentially, to block a backdoor path, one must control for confounders, mediators, or both along the path to prevent the flow of spurious information. However, complications can arise when colliders are involved. In our case, special attention must be given to backdoor paths that include CAPE when using the NOTEARS DAG, due to its varying roles, as discussed in the previous section. Typically, Path 10 is already blocked without additional adjustment because CAPE acts as a collider, naturally blocking information flow along that path^30^. However, to block Path 12, where CAPE functions as the sole confounder, we must condition on CAPE. Doing so inadvertently “opens” Path 10, since conditioning on a collider introduces spurious, non-causal associations between its parent variables (e.g., LCL and ELR), and potentially between their downstream effects (e.g., ARO and ETH). This issue is known as collider bias^46^. To mitigate this, we must re-close Path 10 by additionally conditioning on the other confounders, LCL or ELR.

To explain the collider bias, intuitively, we view CAPE as a function of its parent variables: $\textrm{CAPE} = f(\textrm{LCL}, \textrm{ELR}) + \varepsilon$, where $f(\cdot )$ is an arbitrary function and $\varepsilon$ represents noise term. Conditioning on CAPE implicitly restricts the possible combinations of LCL and ELR that can yield a certain CAPE value. This restriction creates an additional, artificial dependence between LCL and ELR, consequently between ARO and ETH, because knowing one variable influences the expected value of the other. This highlights the risk of controlling for previously-known covariates without explicitly understanding their roles within the underlying causal structure specific to the dataset under investigation.

Note that in CAM-UV, ELR is positioned as a mediator along the ARO–ETH pathway and thus should not be controlled for. Adjusting for ELR would partially or entirely block the causal signal by adjusting for the mechanism through which the effect is transmitted. In essence, this action removes the very pathway we aim to estimate, leading to biased or attenuated causal effect estimates. Therefore, under the assumption that the learned causal graph accurately reflects the true data generation process, only the LCL adjustment is required for the CAM-UV DAG.

### Causal inference estimates average aerosol effects

As another key novelty of our study, we apply a state-of-the-art causal inference framework, Double/Debiased Machine Learning (DML^35^), to estimate the aerosol effects on ETH. DML leverages two ML models of choice to flexibly capture nonlinear relationships, while mitigating bias from both confounding and prediction errors in the ML models themselves.

**Fig. 2:**
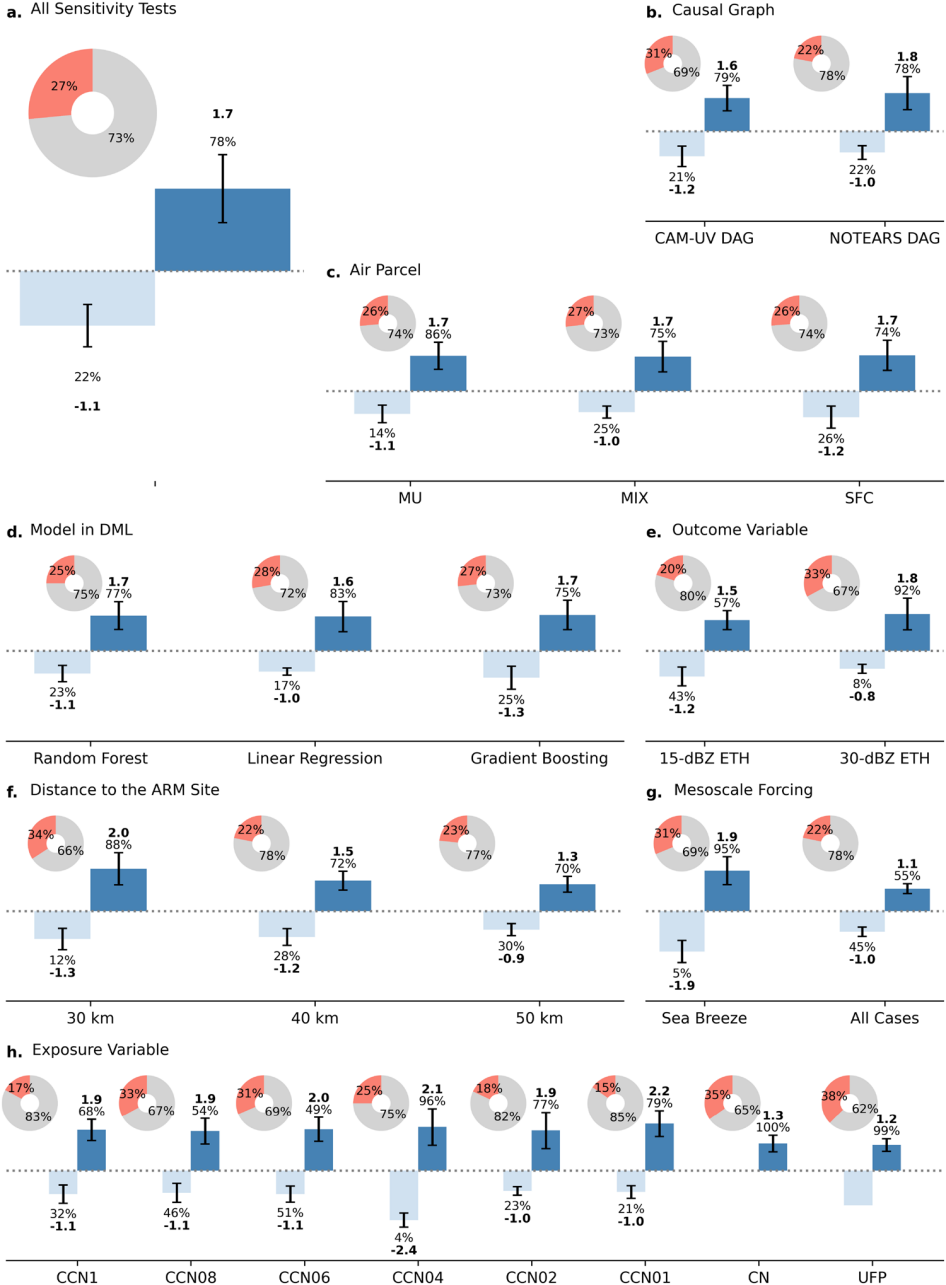
Donut plots show the percentage of valid scenarios across all sensitivity tests (listed in Table S3) in red, and invalid scenarios in gray. Among the valid scenarios, average positive effects are displayed in the bar plots in dark blue with error bars, while negative effects are shown in light blue. The percentages indicated on the bars represent the proportion of positive or negative effects among the valid cases, and the values in bold denote the mean effect estimates (in kilometers). Breakdowns by individual sensitivity tests are presented in each subplot. Within each comparison group, other parameters are held constant.

We performed DML separately on a series of parallel datasets, each representing a different sensitivity test (See Table S2 for details). As shown in Fig. 2, we display the proportion of scenarios where the 95% confidence interval does not include zero, which indicates that the estimated ARO effect is statistically significant (or valid), using donut plots. For these valid scenarios, the mean estimated ARO effects across sensitivity tests are presented in bar plots, separately for positive and negative effects. As shown in Fig. 2a, only a small fraction (27%) of valid scenarios is identified across all sensitivity tests (1,728 in total), highlighting that aerosol has no discernible effect on ETH in the majority of cases. Note that the percentage for ARO–ETH causal link identification (22%, mean result of two models, in previous subsection) is very close to the validation percentage here (27%), providing additional confirmation of the rarity of this relationship. When an effect is detected, the estimates suggest the potential for both invigoration (78%, +1.7 km) and suppression (22%, -1.1 km), with invigoration appearing more likely.

**Table 2:** P-values from two-proportion Z-tests compare the proportion of valid cases between pairs of sensitivity tests, while Mann–Whitney U tests are conducted on valid cases only to assess differences in estimated aerosol effect distributions between the same pairs. To control the family-wise error rate arising from multiple comparisons (e.g., among different aerosol parameter pairs), Bonferroni correction is applied. The reported p-values are adjusted ones. * indicates statistical significance at the 5% level (p-value < 0.05), providing evidence against the null hypothesis. MU = the most unstable parcel; SFC = surface parcel; MIX = mixed-layer parcel.

Comparison (V1 vs V2)	Z-test p-value	Mann–Whitney U p-value
All Case vs Sea Breeze	0.000*	0.000*
CAM-UV DAG vs NOTEARS DAG	0.000*	0.067
15-dBZ ETH vs 30-dBZ ETH	0.000*	0.000*
MU vs SFC	1.000	0.086
MU vs MIX	1.000	0.176
SFC vs MIX	1.000	1.000
Linear Regression vs Random Forest	0.785	1.000
Linear Regression vs Gradient Boosting	1.000	1.000
Gradient Boosting vs Random Forest	1.000	1.000
30 km vs 40 km	0.000*	0.000*
30 km vs 50 km	0.000*	0.000*
50 km vs 40 km	1.000	0.812
N$_{ufp}$ vs N$_{ccn01}$	0.000*	0.007*
N$_{ufp}$ vs N$_{ccn1}$	0.000*	1.000
N$_{ccn06}$ vs N$_{ccn01}$	0.006*	0.017*
N$_{ccn08}$ vs N$_{ccn01}$	0.002*	0.043*
N$_{cn}$ vs N$_{ccn01}$	0.000*	0.017*
N$_{ufp}$ vs N$_{ccn02}$	0.001*	1.000
N$_{ufp}$ vs N$_{ccn04}$	0.332	0.000*
N$_{ccn06}$ vs N$_{ccn1}$	0.037*	1.000
N$_{ccn08}$ vs N$_{ccn1}$	0.014*	1.000
N$_{cn}$ vs N$_{ccn1}$	0.004*	1.000
N$_{ccn04}$ vs N$_{ccn01}$	0.503	1.000
N$_{ccn02}$ vs N$_{ccn01}$	1.000	1.000
N$_{ufp}$ vs N$_{ccn08}$	1.000	1.000
N$_{ufp}$ vs N$_{cn}$	1.000	1.000
N$_{ufp}$ vs N$_{ccn06}$	1.000	1.000
N$_{ccn04}$ vs N$_{ccn1}$	1.000	0.670
N$_{ccn02}$ vs N$_{ccn1}$	1.000	1.000
N$_{ccn04}$ vs N$_{ccn02}$	1.000	0.844
N$_{cn}$ vs N$_{ccn02}$	0.009*	1.000
N$_{ccn08}$ vs N$_{cn}$	1.000	1.000
N$_{ccn08}$ vs N$_{ccn02}$	0.032*	1.000
N$_{ccn08}$ vs N$_{ccn04}$	1.000	0.000*
N$_{ccn06}$ vs N$_{ccn02}$	0.080	0.486
N$_{ccn06}$ vs N$_{ccn04}$	1.000	0.000*
N$_{ccn06}$ vs N$_{cn}$	1.000	1.000
N$_{ccn06}$ vs N$_{ccn08}$	1.000	1.000
N$_{cn}$ vs N$_{ccn04}$	1.000	0.000*
N$_{ccn1}$ vs N$_{ccn01}$	1.000	1.000

A clear contrast emerges when comparing different causal graph assumptions (Fig. 2b). Using the CAM-UV-derived DAG, which identifies only one adjustable covariate (LCL), yields a relatively high validation rate of 31%, compared to 22% when using the NOTEARS DAG with three adjustable covariates. However, the distributions of estimated aerosol effects for valid scenarios are similar between the two approaches (*p* = 0.067, Table 2), both indicating an evident tendency toward aerosol-induced invigoration. This result also indicates that controlling for more covariates does not necessarily dilute the signal but rather enhances its interpretability and contextual validity. This comparison emphasizes the critical role of causal structure specification in guiding robust inference and warns against the biases introduced by neglecting confounders in the identification of aerosol-susceptible scenarios^47^.

Regarding the choice of air parcel for calculating meteorological variables (Fig. 2c), there is insufficient evidence to suggest a significant difference between using three air parcels (Table 2). This is physically consistent with summertime conditions in Houston, where the surface, the most unstable parcel, and the mixed-layer parcel are typically located at similar levels.

Results from the DML framework using three base-learner models (Fig. 2d) show that the fractions of valid cases identified are statistically indistinguishable across models (Table 2). Similarly, the magnitudes of the estimated aerosol effects are also comparable. This indicates that, given the same causal graph, the linearity assumption does not significantly bias the estimates in our particular case. This contrasts with the significant differences observed under different assumed causal graphs in Fig. 2b. On the whole, these findings suggest that linear regression may be suitable for causal inference in certain datasets, but only when the causal structure is well-identified and the relationship is properly deconfounded. Within the valid subsets, however, the fraction of positive effects is slightly higher when using linear regression, suggesting that its reliance on linear assumptions may lead to an over-identification of scenarios as aerosol-invigoration favorable. This may impact case-study-based inference that relies on a scenario flagged solely by linear regression. On a positive note, the consistency of results across models highlights a central strength of the DML framework, which is its resilience to biases introduced by specific base-learner models (see Section 4.3 for more details).

When using alternative proxies for DCC strength–15-dBZ versus 30-dBZ ETH (Fig. 2e), we observe divergent inferred effects. Using the 15-dBZ proxy, 57% of valid cases indicate invigoration (+1.2 km), whereas this percentage increases to 92% (+1.8 km) when using the 30-dBZ proxy. This contrast suggests that aerosol-induced invigoration primarily manifests within the warmer-phase layers of DCCs, as the 30-dBZ ETH typically corresponds to lower, liquid-dominated regions. These findings are broadly consistent with previous studies^48^, which attribute the warm-phase invigoration to enhanced condensation and reduced supersaturation in the low levels of DCC cores.

The vertical layer between the 30-dBZ and 15-dBZ ETHs corresponds to higher altitudes dominated by ice processes; hence, the increased frequency of suppression events identified using the 15-dBZ ETH implies that, in some cases, additional condensate loading and/or dry air entrainment in polluted environments counteract the invigoration observed at lower levels, leading to a lower cloud top or 15-dBZ ETH. Overall, as demonstrated in^12,49^, the net aerosol effect on DCC intensity likely depends on height, reflecting the competing influences of condensate loading, latent heating, and supersaturation. Our results not only provide observational support for the theoretical framework proposed by^12^ but also underscore the risk of drawing inconsistent conclusions when relying on a single, arbitrary proxy for DCC strength.

When investigating DCCs from different distances to the ARM site (see Section 4.1 for more detail, Fig. 2f), we observe an evident trend: both the estimated effect and associated uncertainty decrease as a larger radius is considered. This is expected, as expanding the distance increases the sample size, which in turn enhances statistical stability. In addition, the similarity between 40 km and 50 km (Table 2) suggests that the results begin to stabilize beyond 40 km. However, this comes at the potential cost of reduced representativeness, as meteorological variables may become less reflective of local conditions with increasing distance to the locations of DCC initiation. Despite this trade-off, a consistently higher proportion of invigoration cases is observed across all distances. In contrast, the percentage of valid scenarios is highest for the 30 km scenario, suggesting that a radius of approximately 30 km may represent the effective range of DCC–aerosol interactions around the ARM main site. In addition, this finding provides a useful guideline for determining the representative horizontal scale of single-point measurements under similar meteorological and convective conditions.

Interestingly, when restricting the analysis to sea breeze–associated DCCs, we observe a substantial increase in the proportion of valid cases (31%), with nearly all (95%) indicating aerosol invigoration effects (Fig. 2g). The estimated average effect reaches as high as 1.9 km, which is 0.8 km greater than the estimate derived from the full DCC sample. The differences are statistically significant (*p* = 0.000, Table 2). This enhancement is likely driven by the dual role of the sea breeze front where it not only serves as a trigger for DCC initiation but also facilitates aerosol uplift along the frontal inflow, promoting their interaction with DCC microphysics and dynamics^50^. However, this may also reflect a natural separation between clean versus polluted conditions and between strong versus weak DCCs associated with the passage of the sea-breeze front. More specifically, along the sea-breeze front, DCCs tend to be stronger due to the additional mesoscale forcing, compared to those that develop after the front has passed the same location^51^. The aerosol conditions sampled before the arrival of the sea breeze are often more polluted than those after its passage, when the local air mass is more strongly influenced by marine air^52^. Therefore, further investigation, likely through numerical model simulations, is needed to better clarify the role of the sea breeze in modulating both aerosol and convective properties.

Figure 2h presents results from sensitivity tests in which individual aerosol parameters are varied as exposure variables in the DML framework. An evident feature is that nearly all estimated effects are positive when using $N_{cn}$ (number concentration of aerosol particles between 10 and 3000 nm) and $N_{ufp}$ (number concentration of aerosol particles between 3 and 3000 nm) as exposure variables. Moreover, these two parameters yield the highest fraction of aerosol-sensitive scenarios compared to $N_{ccn}$ measured at different supersaturation levels. This is consistent with the higher correlation coefficients between $N_{cn}$ / $N_{ufp}$ and ETH as shown in Fig. S5. Between $N_{cn}$ and $N_{ufp}$, however, the fraction of aerosol-sensitive cases and the corresponding changes in ETH are nearly identical, indicating that the inclusion of ultrafine particles does not necessarily enhance the inferred invigoration effect under the environmental conditions of this study. It is important to note that these aerosol properties were measured at the surface and are used here solely to distinguish DCCs developing in relatively clean versus polluted conditions. The activation status of these particles within convective updrafts remains unknown due to the lack of direct measurements. Consequently, the processes or pathways through which aerosol particles modulate latent heat release and subsequently influence convective invigoration cannot be determined from the available observations.

Interestingly, unlike $N_{cn}$ and $N_{ufp}$, when other aerosol variables (CCN number concentrations listed in Fig. 2h) are used, the estimated effects span both positive and negative values. Notably, the fraction of aerosol-sensitive cases and the strength of the invigoration or suppression effects do not vary linearly with increasing supersaturation levels. For example, $N_{ccn08}$ and $N_{ccn06}$ yield the highest percentage of aerosol-sensitive scenarios, even though they correspond to neither the highest nor the lowest supersaturation levels used. Both parameters also exhibit a nearly equal distribution between invigoration and suppression scenarios, whereas other $N_{ccn}$ variables tend to show a greater fraction of invigoration scenarios. This result reflects the fact that the classification of polluted versus clean conditions depends strongly on the specific aerosol property used for separation. This highlights potential biases in studies that rely on a single aerosol parameter, as different parameters could yield distinct DCC responses (e.g., in Fig. 2h), and calls for advanced instrumentation capable of measuring supersaturation in updrafts to ultimately verify the warm-phase invigoration mechanism.

In summary, our application of the backdoor criterion and the DML framework enabled a robust estimation of aerosol impacts on ETH, effectively addressing our second research objective. The analysis suggests that increased aerosol concentrations are more likly to enhance DCC intensity, particularly in the warmer phase of DCCs, under sea breeze conditions, and/or when smaller aerosol particles are considered.

## Discussion

To reduce uncertainties in Earth system prediction, identifying the fingerprint of aerosol impacts on DCCs remains a key priority for constraining aerosol–cloud interaction processes in models. To this end, we introduce a novel application of a causal discovery-inference framework and advocate for the use of causal reasoning to more rigorously evaluate aerosol effects on DCCs. The following texts begin with a summary of the principal findings, followed by a discussion of their implications and related aspects of the study.

Leveraging observations from TRACER, we successfully reconstruct the underlying causal relationships among ARO, ETH, and meteorological variables. The resulting causal graphs reveal a direct ARO $\rightarrow$ ETH link in 10% to 35% of cases, suggesting a weak and nonlinear relationship. Additional, multiple plausible causal structures highlight the complex, interactive nature of aerosol–convection–environment processes.

Then, we estimate the aerosol average effect across various sensitivity scenarios using causal inference. The estimates indicate that ARO-impacted scenarios remain infrequent (27%), showing both invigoration and suppression effects. Invigoration is more common under sea-breeze conditions (95%, +1.9 km) and when total aerosol number concentration is used as the exposure variable (>99%, up to +1.3 km), with stronger signals in the warmer phase. Considering possible mediator roles of meteorological variables (as identified by CAM-UV) increases the proportion of aerosol-sensitive scenarios to 31%.

Although the central challenges have been addressed, several caveats remain. For instance, it is possible that some important confounding factors have not yet been discovered or cannot be directly measured, introducing possible uncertainty into the analysis. One such factor could be boundary layer updraft velocity, which may play a critical role in sea breeze scenarios by modifying DCC intensity and aerosol distributions. However, this variable cannot be controlled for to verify this hypothesis, as relevant measurements are not available for all DCC cases. Fortunately, the impact of unmeasured variables is not always detrimental. In a situation where an unobserved confounder lies on a backdoor path that is already blocked by controlling for other variables, its absence does not bias the estimation at all. Other techniques, such as front-door adjustment and instrumental variable methods^30^, can also yield unbiased estimates without the need to block backdoor, confounded paths. This highlights the elegance and practical power of thinking in terms of causal graph theory. This is in contrast to conventional approach, where one may feel compelled to include as many potentially relevant variables as possible, assuming that omission always leads to bias.

Nonetheless, future studies would benefit from more comprehensive measurements of both convection and its surrounding environment, ideally covering multiple sites across the region with higher temporal resolution. For instance, a network of sounding launches could capture the spatial heterogeneity of thermodynamic conditions, allowing for a more accurate characterization of the environment associated with each tracked convective cell. Additionally, in-cloud measurements of vertical velocity and supersaturation are highly valuable and essential for directly evaluating the various invigoration hypotheses.

Although our study focused on aerosol–convection interactions in the Houston region, the results may be generalizable, to some extent, to other coastal megacities where similar aerosol species and number concentrations, cloud types, meteorological conditions, and geographical configurations prevail. However, the unique characteristics of each region or city can potentially modulate the susceptibility of DCCs to aerosol perturbations and therefore warrant further investigation. Factors such as city size, proximity and orientation relative to the coastline, multiscale circulation interactions, and prevailing climate conditions can all influence how aerosols affect convective development in different environments.

During the summer months in the Houston region, the predominant aerosol species are organic (49%) and sulfate (34%)^34^. For regions with different aerosol compositions or climatic conditions, further analyses can be conducted using our causal framework, with an appropriately tailored set of confounders selected to reflect the unique characteristics of each study location.

Another frequently asked question in observational studies (especially those employing machine learning techniques) is what constitutes an optimal sample size for addressing a given scientific question. Determining such an optimal sample size is inherently challenging in our study, as no ground truth exists for evaluating the accuracy of the inferred causal structures. Moreover, the ability to reveal a robust causal relationship can depend on several additional factors, including the degree of linearity in the underlying relationships, whether all relevant confounding variables are adequately accounted for, and how well the available measurements represent the key physical processes of interest. These complexities make it difficult to define a universal or problem-specific optimal sample size.

In our analysis, we indirectly assessed the influence of sample size by comparing DCCs collected within different distances from the ARM site (Fig. 2f). Although the quantitative results vary somewhat, the qualitative conclusions remain consistent - the predominance of aerosol invigoration effects. While other factors beyond sample size likely contribute to these variations, this consistency, to some extent, indicates that our results are not substantially biased by limited sample availability. Nevertheless, extending the observation period beyond the TRACER campaign would be valuable for further validating these findings and exploring related scientific questions.

Overall, our findings emphasize the nuanced and context-dependent nature of aerosol impacts on DCCs and caution against broad generalizations based on limited scenarios. They also underscore the essential role of establishing causal links when interpreting observational data in atmospheric science more broadly. By revealing the underlying causal relationships, our findings offer valuable constraints for the development and improvement of next-generation convection-permitting models. In addition, the proposed causal framework provides a powerful tool for advancing machine learning applications in atmospheric science, facilitating data-driven model evaluation and informing the design of physics-aware parameterizations.

## Methods

### Datasets for causal analyses

As the first step in the analysis, we use an open-source Lagrangian cell tracking algorithm developed by^53^ to follow the lifecycle of DCC rainfall cores and quantify their maximum 30-dBZ and 15-dBZ ETH. Specifically, we input Level-II reflectivity data from the NOAA S-band Doppler radar KHGX-Houston^54^ at the 2-km altitude. The radar data, gridded at 1-km horizontal resolution, cover a 400 $\times$ 400 km domain centered on the radar site (29$^{\circ }$28’19”N, 95$^{\circ }$04’45”W). Note that all samples at different elevation angles are used in the regridding process. Due to the increasing radar beam width with distance from the radar center, the gridded data have relatively lower spatial resolution at remote locations compared to those closer to the radar, which may affect the estimation of reflectivity and ETH for cells farther from the radar. Rainfall cores are identified as contiguous regions where 2-km radar reflectivity exceeds 10 dBZ, with a peak value greater than 40 dBZ and a maximum 30-dBZ ETH of at least 5 km above ground level at some point during their evolution^55^. These rainfall cores were all developed over land, as shown in^34^ (their Fig. 5). Additional tracking details, including preprocessing steps, selection criteria, and cell characteristics can be found in^34,51^.

We restrict our analysis to days characterized by anticyclonic regimes in 2022^51^ to exclude DCCs dominated by strong synoptic-scale forcing, under which aerosol impacts may be overwhelmed and difficult to detect. These regimes are identified using a self-organizing map (SOM) approach^56^ trained on geopotential height anomalies at 700 hPa from the reanalysis data^57^. The detailed methodology and results are provided in^51,58^.

We quantify the pre-convective meteorological conditions for tracked DCCs using ARM balloon-borne sounding system (SONDE) observations^59^. Following parcel theory, we derive a suite of key thermodynamic and dynamic variables documented to influence aerosol-convection interactions^15,28,60,61^. Specifically, eight candidate variables are considered: CAPE, LCL, LNB, ELR, convective inhibition (CIN), level of free convection (LFC), low-level wind shear between the surface and 5 km (WSR), and mean relative humidity below 5 km (RH), calculating for three different parcel types: mixed-layer (MIX), most-unstable (MU), and surface (SFC). The SFC parcel is defined as the parcel at the lowest level of the sounding data; the MU parcel is defined as the parcel with the greatest virtual temperature within the lowest 700 mb above the surface; and the MIX parcel is defined as the parcel with thermodynamic properties averaged over the boundary layer^22^. The parcel is assumed to rise pseudo-adiabatically without entrainment, with hydrometeor loading effects neglected, following^22^.

Aerosol conditions prior to DCC initiation are characterized using surface-based measurements from the ARM Aerosol Observing System (AOS^62^). As part of our sensitivity analyses, we consider eight different aerosol metrics as exposure variables. These include: CCN number concentrations at six supersaturation setpoints: 0.1% ($N_{ccn01}$), 0.2% ($N_{ccn02}$), 0.4% ($N_{ccn04}$), 0.6% ($N_{ccn06}$), 0.8% ($N_{ccn08}$), and 1% ($N_{ccn1}$); Total aerosol number concentration for particles with diameters between 10 and 3,000 nm ($N_{cn}$) and between 3 and 3,000 nm ($N_{ufp}$, including ultrafine particles). $N_{ccn}$ at various supersaturations was measured using a dual-column CCN counter, $N_{cn}$ was measured using a fine-mode condensation particle counter, and $N_{ufp}$ was measured using an ultrafine condensation particle counter. The $N_{cn}$ and $N_{ufp}$ measurements had a temporal resolution of 1 min, while $N_{ccn}$ at various supersaturations was measured twice per hour. Each of these metrics is tested individually in our causal analyses to assess their specific impacts.

Based on these measurements, we construct a case-based dataset^63^(86 samples) to serve as inputs for the causal models. Each data point is constructed by pairing meteorological variables from individual radiosondes, aerosol properties averaged over a 1-hour window centered on the sounding launch time, and mean DCC ETHs observed within 6 hours after the launch and within a 50 km radius of the ARM main site. Alternative thresholds were tested, including adjustments to DCC selection criteria based on the maximum distance from the ARM main site (i.e., 30 km [61 samples], 40 km [70 samples]) and the presence of sea breezes (identified in^51^), as the arrival of sea breezes can modulate both convective and aerosol characteristics over the region^51,52^.

To facilitate causal analysis, aerosol data are binarized by assigning a value of 0 to measurements below the median and 1 to those above, representing clean and polluted conditions, respectively. As a result, the causal estimates of mean aerosol effects on ETH are expressed in kilometers. This approach enhances result interpretability and ensures applicability to other locations, independent of regional differences in baseline aerosol conditions.

All other continuous variables are standardized by applying a z-score normalization (mean = 0, standard deviation = 1) to ensure comparability across variables and to prevent bias toward features with larger numeric ranges such as CAPE.

Some caveats are worth noting regarding the dataset used in this study. The aerosol measurements represent only surface conditions, as in-cloud aerosol observations were not collected during the field campaign. Radiosondes were launched 4–7 times per day, therefore, the measurements may not fully capture the exact pre-convective environment for all events as they evolve over time. Additionally, the NEXRAD radar has a temporal resolution of approximately 5 minutes, which may not always coincide with the peak ETH during the DCC lifecycle, potentially introducing sampling biases.

### Causal discovery models

Two nonlinear causal discovery models , CAM-UV^38^ and NOTEARS^39^, are employed to uncover the underlying causal structure in the observational data, each offering complementary strengths. Both models represent state-of-the-art methodologies and have demonstrated robust performance across diverse fields such as epidemiology^64^, biology^65^, and social science^66^; however, their application in atmospheric science remains novel.

Note that both models assume that the data-generating process follows a DAG, which implies that the underlying causal structure is acyclic, containing no cycles or feedback loops. While this assumption may appear limiting, particularly given that many atmospheric processes involve feedback operating over various timescales, it can still be appropriate when analyzing relationships within a constrained temporal window. In our study, we focus on short-term interactions associated with DCC development, where the dominant causal influences can be considered locally acyclic. Within this timescale, DAG-based models remain applicable and effective^22^.

CAM-UV is chosen for its solid mathematical foundations, intuitive interpretability, suitability for case-based datasets like ours, and more importantly, the ability to detect potential unobserved confounders. It infers causal directions by exploiting inherent ”asymmetries” in the data-generating process, an attribute commonly observed in real-world systems, including those in atmospheric science^67^. In essence, the true causal mechanism leaves identifiable traces when two variables are nonlinearly related. Specifically, residuals from the correctly specified causal model ($y=f(x)+\epsilon _1$, $x \rightarrow y$) tend to be approximately independent of the predictor or the hypothesized cause (*x*), whereas residuals from the anti-causal model ($x=f(y)+\epsilon _2$, $y \rightarrow x$) often display substantial dependence of the predictor (*y*) (see Text S1.1 in the supplemental material for an example).

CAM-UV belongs to the functional causal discovery model family and builds upon the Additive Noise Model (ANM) framework. Let $X = (X_1, \ldots , X_d)$ denote a random vector. CAM-UV models each variable, $X_j$, as a nonlinear function of its parents, $X_{pa}$, plus an additive, independent noise term, $N_j$:

1 $$\begin{aligned} X_j = f_j(X_{\text {pa}(j)}) + N_j, \quad N_j\perp \!\!\!\perp f_j(X), \quad \mathbb {E}[f_j(X)] = 0 \end{aligned}$$

where $f_j(\cdot )$ is a Generalized Additive Model^68^, inferring nonlinearity by fitting flexible smooth functions to the data, *d* is the number of variables or the dimension of the causal graph. The condition $\mathbb {E}[f_j(X)] = 0$ is imposed to mean-center $f_j$, thereby fixing the scale and ensuring that the model is uniquely identifiable given the data.

CAM-UV follows three main steps: First, it identifies candidate parents for each variable by evaluating subsets of variables. In each subset, it selects the sink variable–the one most statistically independent from the others. This independence is tested using a nonparametric independence test, the Hilbert–Schmidt Independence Criterion, applied to residuals from $f_j(\cdot )$. The remaining variables are added to its candidate parent set. Second, CAM-UV refines these candidate sets by removing any variables whose residuals remain independent after conditioning on the rest, leaving only the true parents for each variable. The final step represents a key novelty of the CAM-UV model, its ability to detect potential unobserved confounders. CAM-UV examines mutual dependence between the residuals of different variables after regressing out their identified parents. If residuals remain dependent, this suggests the presence of a hidden common cause, and CAM-UV flags the corresponding edge as ‘NaN’. In this study, we omit these ‘NaN’ edges in order to eliminate spurious relationships and focus on interpretable directed connections.

Unlike CAM-UV, which relies on additive noise model assumptions, nonlinear NOTEARS accommodates nonlinear dependencies among variables without requiring explicit specification of their functional forms, making it well-suited for modern machine learning applications and a broader range of data structures. Despite its modeling flexibility, nonlinear NOTEARS does not rely on independence tests; instead, it reformulates the problem of DAG structure learning as a continuous optimization task by introducing a smooth acyclicity constraint, enabling scalable, differentiable, and flexible causal discovery.

In essence, NOTEARS is a score-based causal discovery model that computes a numerical summary to quantify how well a candidate causal graph fits the observed data in the optimization process. The objective is to recover the DAG structure under the assumption that the data are generated according to a Structural Equation Model:

2 $$\begin{aligned} \mathbb {E}[X_j \mid X_{\text {pa}(j)}] = f_j(X), \quad \mathbb {E}[f_j(X)] = 0 \end{aligned}$$

where $\mathbb {E}[X_j \mid X_{\text {pa}(j)}]$ is a conditional expectation, meaning the expected value of $X_j$ given its parents $X_{\text {pa}(j)}$. $f_j: \mathbb {R}^d \rightarrow \mathbb {R}$ is the core functional relationship between the variable $X_j$ and its candidate parent variables $X_\text {pa}(j)$. In this study, $f_j$ are multilayer perceptrons^69^, a feedforward neural network consisting of fully connected neurons with nonlinear activation functions.

The key is to minimize the prediction error of $f_j$ or the empirical loss while ensuring the resulting causal graph (*G*) is acyclic and sparse. Given *n* i.i.d. samples $\{x^{(i)}\}_{i=1}^n$, the empirical loss is defined as:

3 $$\begin{aligned} \mathcal {L}(f) = \frac{1}{n} \sum _{j=1}^d \sum _{i=1}^n \ell \left( x_j^{(i)}, f_j(x^{(i)})\right) \end{aligned}$$

where $\ell (\cdot , \cdot )$ is a loss function (e.g., squared loss). The goal is to solve:

4 $$\begin{aligned} \min _f \; \mathcal {L}(f) \quad \text {subject to} \quad G(f) \in \text {DAG} \end{aligned}$$

The condition $G(f) \in \text {DAG}$ enforces that the learned causal graph must be acyclic. A major innovation of NOTEARS is its avoidance of combinatorial cycle-checking, as required in traditional score-based methods. Instead, it introduces a continuous and differentiable acyclicity constraint:

5 $$\begin{aligned} h(W(f)) = \operatorname {tr}\left( e^{W(f) \circ W(f)}\right) - d = 0 \end{aligned}$$

where $\circ$ denotes the element-wise (Hadamard) square to ensure non-negative values and smoothness for optimization. $tr(\cdot )$ is a trace operator, which sums the main diagonal elements of a matrix. In this context, $tr(\cdot )$ quantifies the “total weight of cycles” in the causal graph. An example to demonstrate this acyclicity constraint is provided in the supplemental material (Text S1.2).

$W(f) \in \mathbb {R}^{d \times d}$ in Eq. (5) is the functional dependency matrix or nonparametric adjacency matrix, indicating the causal strength among variables. It is captured by the partial derivative:

6 $$\begin{aligned} [W(f)]_{kj} = \left\| \frac{\partial f_j}{\partial X_k} \right\| _{L^2} \end{aligned}$$

where $\frac{\partial f_j}{\partial X_k}$ evaluates how strong variable $X_k$ affects variable $X_j$^70^. The value $[W(f)]_{kj}$ becomes the (*k*, *j*) entry of the dependency matrix *W*(*f*). The notation $\left\| \cdot \right\| _{L^2}$ refers to the $L^2$-norm, known as the root-mean-square (RMS) value.

Combining the empirical loss and acyclicity, the final objective of NOTEARS becomes:

7 $$\begin{aligned} \min _{f_j \in H^1(\mathbb {R}^d)} \; \mathcal {L}(f) + \lambda \sum _{j=1}^d \sum _{k \ne j} \left\| \frac{\partial f_j}{\partial X_k} \right\| _{L^2} \quad \text {subject to} \quad h(W(f)) = 0. \end{aligned}$$

where $H^1$ is a Sobolev space ensuring the partial derivatives exist and are square-integrable. Note that a regularization term is introduced to penalize the total strength of functional dependencies between variables to encourage sparsity in the learned causal graph. For example, if $\frac{\partial f_j}{\partial X_k}$ is near zero, indicating that $f_j$ remains nearly invariant with respect to changes in $X_k$, then $X_k$ is unlikely to be a direct cause of $X_j$, and no edge is needed between the two. $\lambda$ is set to 0.01 in our study.

Overall, NOTEARS enables flexible, data-driven causal discovery in systems where nonlinear and potentially high-dimensional interactions are expected like in aerosol-convection-environment interactions. It outperforms several leading causal discovery models when evaluated on nonlinear synthetic datasets^39^.

To mitigate the possible uncertainty of the one-time learned causal graph, we use a bootstrap procedure. Specifically, 100 resampled datasets are generated from the original data for each sensitivity test (576 tests, in Table S1), and the causal discovery models are applied to each. We then report the proportion of times each directed edge is identified across all runs (57,600 in total), with higher proportions indicating greater plausibility of the corresponding causal directions. Note that bidirectional relationships between variable pairs may be identified across separate model runs (e.g., LCL $\rightarrow$ ELR for 65%, ELR $\rightarrow$ LCL for 30%); however, we report only the direction that occurs most frequently (LCL $\rightarrow$ ELR).

### Double/Debiased machine learning

We estimate the aerosol effects on ETH by using the DML model^35^, which enables the use of nonlinear machine learning methods for deconfounding. DML also guards against biases introduced by base learner prediction errors through a structured three-step procedure.

In the first step, we predict the part of exposure (*D*) and outcome (*Y*) that is due to covariates (*X*) by fitting two ML models, also called the nuisance function. The first model is an estimated conditional expectation of *Y* given *X* based on our data and can be expressed as:

8 $$\begin{aligned} \hat{m}(x) \approx \mathbb {E}[Y \mid X] \end{aligned}$$

In the second model, we estimate the conditional expectation of *D* given *X*:

9 $$\begin{aligned} \hat{p}(x) \approx \mathbb {E}[D \mid X] \end{aligned}$$

In our case, Eq. (8) refers to what would the ETH be given meteorological variables (identified to block backdoor paths), regardless of aerosol conditions. Equation 9 estimates what aerosol condition would be only given meteorological variables. This step builds a baseline world: What does the meteorological conditions usually impact ETH and aerosols separately.

In the second step, we compute two residuals ($\tilde{Y}$ and $\tilde{D}$) to get the part of the *Y* and *D* not explained by the meteorological conditions, respectively. In other words, this is for “removing” the influence of the meteorological factors from *Y* and *D*.

10 $$\begin{aligned} & \tilde{Y} = Y - \hat{m}(X) \end{aligned}$$

11 $$\begin{aligned} & \tilde{D} = D - \hat{p}(X) \end{aligned}$$

In the final step, we perform a residual-on-residual regression, that is to say, we estimate the causal effect $\tau$ of *D* on *Y* from residuals by regressing $\tilde{Y}$ on $\tilde{D}$ using simple linear regression, $\tilde{Y}_i = \hat{\tau } \cdot \tilde{D}_i + \epsilon _i$. Therefore,

12 $$\begin{aligned} \hat{\tau } = \frac{\mathbb {E}[\tilde{Y} \cdot \tilde{D}]}{\mathbb {E}[\tilde{D}^2]} \end{aligned}$$

where the numerator represents the expected covariance between the residuals, and the denominator is the expected variance of $\tilde{D}$. This equation quantifies how much of the variation in *Y* can be explained by *D* after removing the influence of *X*. In our context, this translates to: Given predictions of ETH based on meteorology alone, does the remaining variation in aerosols explain any additional differences in ETH? If $\tilde{Y}$ and $\tilde{D}$ are uncorrelated, the numerator will be zero, indicating that *D* has no causal effect on *Y* (i.e., aerosols have no effect on ETH).

Note that this $\hat{\tau }$ estimator is orthogonal to or robust against small errors arising from imperfect ML estimate of $\hat{m}(X)$. This stems from the fact that, although $\tilde{Y}$ may contain model error from $\hat{m}(X)$, the cross-product $\mathbb {E}[\tilde{Y} \cdot \tilde{D}]$ remains unbiased because $\tilde{D}$ is orthogonal to any function of *X* within the learner’s hypothesis space, including the model error. In other words, since $\tilde{D}$ captures the component of *D* that is not explained by *X*, it is uncorrelated with errors that are functions of *X*. This property–known as the Neyman orthogonality condition–is the key to why DML remains robust to imperfect ML model estimation. These are key to successfully dealing with high-dimensional, nonlinear confounding, where traditional causal inference models struggle or overfit.

Sensitivity tests are conducted by fitting Eqs. (8) and (9) using Random Forest^71^ (30 estimators), Gradient Boosting^72^ (30 estimators, learning rate = 0.1, max depth = 3), or Multiple Linear Regression models. Cross-fitting is used in DML to further reduce overfitting. We split the data into 4 folds, and use one fold to estimate $\hat{m}(X)$ and $\hat{p}(X)$, and another to compute $\tilde{D}$, $\tilde{Y}$, and $\hat{\tau }$, then rotate folds to use the full dataset. This prevents “double-dipping”, where the same data is used for both learning and inference.

## Supplementary Information

Supplementary Information.

## Data Availability

Data availability. The authors declare that the processed data supporting the findings of this study are available at https://zenodo.org/records/14298966. In addition, the NEXRAD raw data used for convective cell tracking is accessible at https://registry.opendata.aws/noaa-nexrad/. The SOM synoptic regime classification (SYNOPWEAREG) is available at https://adc.arm.gov/discovery/#/results/instrument_class_code::synopweareg. The meteorological variables calculated from ARM soundings (SONDEPARAM) are available at https://adc.arm.gov/discovery/#/results/instrument_class_code::sondeparam. Aerosol measurements can be downloaded from https://adc.arm.gov/discovery/#/results/primary_meas_type_code::ccn.

## Code availability

The CAM-UV model codes are available at https://lingam.readthedocs.io/en/latest/tutorial/camuv.html. The NOTEARS model codes are available at https://github.com/xunzheng/notears. The Double/Debiased Machine Learning codes are available at https://github.com/py-why/dowhy. The convective cell tracking codes can be found at https://github.com/openradar/TINT.
