# Supplementary material for: Causal machine learning uncovers conditions for convective intensification driven by organic and sulfate aerosols
Source: Sci Rep. 2025 Dec 29;15:44806. doi: 10.1038/s41598-025-28939-x (PMC12749776; doi:10.1038/s41598-025-28939-x)
Supplement: Supplementary file 1 — Supplementary Information. [file 41598_2025_28939_MOESM1_ESM.pdf]

Supplemental Materials for "Causal Machine Learning Uncovers Conditions for Convective Intensification Driven by Organic and Sulfate Aerosols"

Dié Wang[1,2*], Jie Xi Li[3] and Jun Lu[4]

[1*]Environmental Science and Technologies Department, Brookhaven National Laboratory, 98 Rochester St, Upton, 11937, NY, USA.
[2]Institute for Atmospheric and Climate Science, ETH Zurich, Universitätstrasse 16, Zurich, 8092, Switzerland.
[3]Applied Mathematics and Statistics, Stony Brook University, 100 Nicolls Road, Stony Brook, 11794, NY, USA.
[4]School of Public Health, University of Illinois Chicago, 1200 West Harrison Street, Chicago, 60607, IL, USA.

*Corresponding author(s). E-mail(s): die.wang@env.ethz.ch;
Contributing authors: jessieli358@gmail.com; jlu56@uic.edu;

1 Texts

1.1 Non-linearity leaves trace

CAM-UV is a causal discovery model that leverages the fact that when two variables are non-linearly related, the causal mechanism does leave traces.

We first generate some non-linear synthetic data following equations:

$$x := \epsilon_1; y := x^3 + \epsilon_2 \tag{1}$$

where ϵ_1 and ϵ_1 are random noises.

We then fit two non-linear spline regressions to the data with one following the causal direction $x \to y$ and one following the anti-causal direction $y \to x$. As shown in Figure 1 below, the two lines are significantly different. Only the causal fitting well represent the true data distribution.

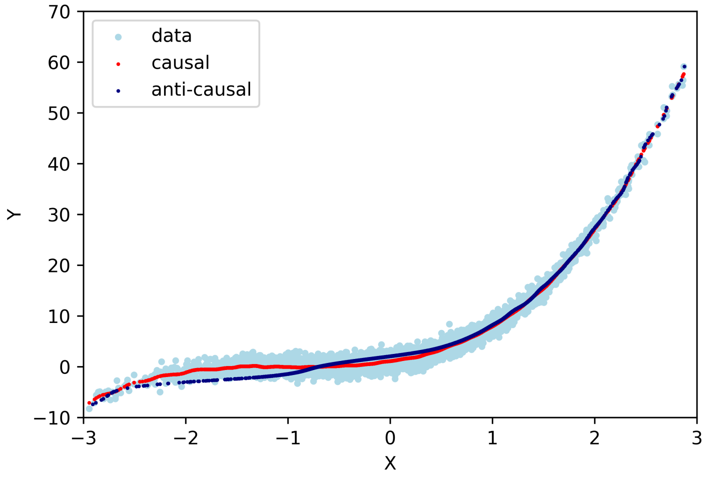

Fig. 1 Scatter plot of non-linear synthetic data with two fitted regression curves

In addition, we compute the residuals for both models and plot them against the predictors. Distinct patterns are evident in Figure 2 below. In particular, the residuals from the anti-causal fit exhibit dependence on the predictor, whereas the residuals from the causal fit appear independent of it.

Therefore, it remains possible to infer the causal direction in the nonlinear dataset by evaluating whether the residual term is independent of the hypothesized cause.

1.2 Examples of acyclicity constraint in NOTEARS

The defined acyclicity constraint is:

$$h(W) = \text{tr}\left(e^{W \circ W}\right) - d \tag{2}$$

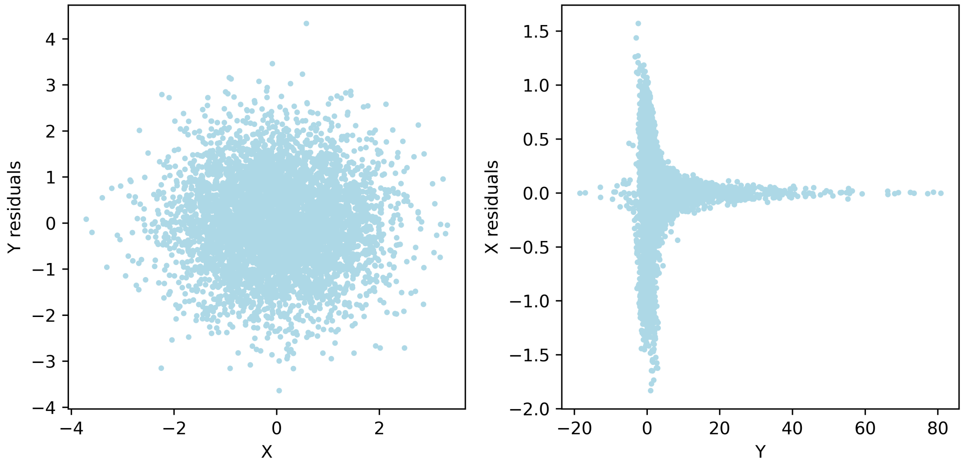

Fig. 2 Scatter plots of residuals for the causal and anti-causal fittings using non-linear synthetic data

1.2.1 An acyclicity case

To test the acyclicity constraint, we first define a simple DAG with 3 nodes where $X_1 \to X_2$ and $X_2 \to X_3$. This is a typical acyclic graph. The corresponding weighted adjacency matrix is:

$$W = \begin{bmatrix} 0 & 1 & 0 \\ 0 & 0 & 1 \\ 0 & 0 & 0 \end{bmatrix} \tag{3}$$

Second, we compute the Hadamard (element-wise) square:

$$A = W \circ W = \begin{bmatrix} 0 & 1 & 0 \\ 0 & 0 & 1 \\ 0 & 0 & 0 \end{bmatrix} \circ \begin{bmatrix} 0 & 1 & 0 \\ 0 & 0 & 1 \\ 0 & 0 & 0 \end{bmatrix} = \begin{bmatrix} 0 & 1 & 0 \\ 0 & 0 & 1 \\ 0 & 0 & 0 \end{bmatrix} \tag{4}$$

Third, we compute the matrix exponential $e^{W \circ W} = e^A$. By definition,

$$e^A = \sum_{n=0}^{\infty} \frac{A^n}{n!} = I + A + \frac{A^2}{2} = \begin{bmatrix} 1 & 0 & 0 \\ 0 & 1 & 0 \\ 0 & 0 & 1 \end{bmatrix} + \begin{bmatrix} 0 & 1 & 0 \\ 0 & 0 & 1 \\ 0 & 0 & 0 \end{bmatrix} + \frac{1}{2} \begin{bmatrix} 0 & 0 & 1 \\ 0 & 0 & 0 \\ 0 & 0 & 0 \end{bmatrix} = \begin{bmatrix} 1 & 1 & 0.5 \\ 0 & 1 & 1 \\ 0 & 0 & 1 \end{bmatrix} \tag{5}$$

Fourth, we take the trace which is equal to the sum of the square matrix eigenvalues, and then we compute $h(W)$:

$$\mathrm{tr}(e^A) = 1 + 1 + 1 = 3 \quad \Rightarrow \quad h(W) = \mathrm{tr}(e^A) - 3 = 0 \tag{6}$$

This confirms that the causal graph is acyclic.

1.2.2 A cyclic case

Now we assume a weighted adjacency matrix as:

$$W = \begin{bmatrix} 0 & 1 & 0 \\ 0 & 0 & 1 \\ 1 & 0 & 0 \end{bmatrix} \tag{7}$$

In this case, the matrix has a cycle or loop: $X_1 \to X_2 \to X_3 \to X_1$.
The Hadamard (element-wise) square is:

$$A = W \circ W = \begin{bmatrix} 0 & 1 & 0 \\ 0 & 0 & 1 \\ 1 & 0 & 0 \end{bmatrix} \circ \begin{bmatrix} 0 & 1 & 0 \\ 0 & 0 & 1 \\ 1 & 0 & 0 \end{bmatrix} = \begin{bmatrix} 0 & 1 & 0 \\ 0 & 0 & 1 \\ 1 & 0 & 0 \end{bmatrix} \tag{8}$$

Note that e^A is a cyclic permutation matrix with the property:

$$A^0 = I, \quad A^1 = A, \quad A^2 = A^2, \quad A^3 = I, \quad A^4 = A, \quad A^5 = A^2, \quad \text{etc.}$$

Using the periodicity $A^3 = I$, we can group terms by powers of 3:

$$e^A = \sum_{k=0}^{\infty} \frac{A^{3k}}{(3k)!} + \sum_{k=0}^{\infty} \frac{A^{3k+1}}{(3k+1)!} + \sum_{k=0}^{\infty} \frac{A^{3k+2}}{(3k+2)!}$$

Since:

$$A^{3k} = I, \quad A^{3k+1} = A, \quad A^{3k+2} = A^2,$$

we write:

$$\exp(A) = f_0 I + f_1 A + f_2 A^2$$

where:

$$f_0 = \sum_{k=0}^{\infty} \frac{1}{(3k)!}, \quad f_1 = \sum_{k=0}^{\infty} \frac{1}{(3k+1)!}, \quad f_2 = \sum_{k=0}^{\infty} \frac{1}{(3k+2)!}$$

This gives:

$$e^A \approx f_0 \begin{bmatrix} 1 & 0 & 0 \\ 0 & 1 & 0 \\ 0 & 0 & 1 \end{bmatrix} + f_1 \begin{bmatrix} 0 & 1 & 0 \\ 0 & 0 & 1 \\ 1 & 0 & 0 \end{bmatrix} + f_2 \begin{bmatrix} 0 & 0 & 1 \\ 1 & 0 & 0 \\ 0 & 1 & 0 \end{bmatrix}$$

Using a few terms from the Taylor series, we approximate:

$$f_0 \approx 1 + \frac{1}{6!} + \frac{1}{9!} \approx 1.0014,$$

$$f_1 \approx \frac{1}{1!} + \frac{1}{4!} + \frac{1}{7!} \approx 1.0420,$$
$$f_2 \approx \frac{1}{2!} + \frac{1}{5!} + \frac{1}{8!} \approx 0.5436.$$

Therefore, the matrix exponential can be approximated as:

$$e^A \approx \begin{bmatrix} 1.0014 & 1.0420 & 0.5436 \\ 0.5436 & 1.0014 & 1.0420 \\ 1.0420 & 0.5436 & 1.0014 \end{bmatrix}$$

We will get:

$$\mathrm{tr}(e^{W \circ W}) > 3 \Rightarrow h(W) > 0 \tag{9}$$

It is evident that the constraint is violated and that we correctly detect a cycle.

2 Figures and Tables

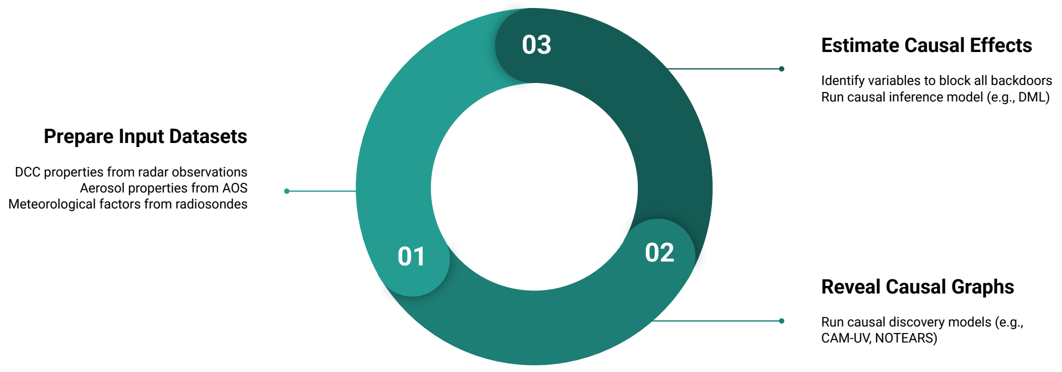

Fig. 3 Schematic overview of the methodology.

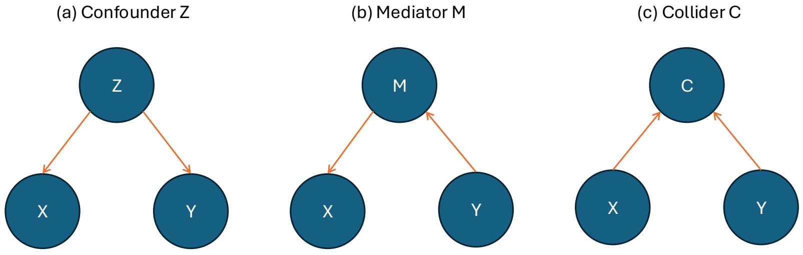

Fig. 4 Terminology in causal inference.

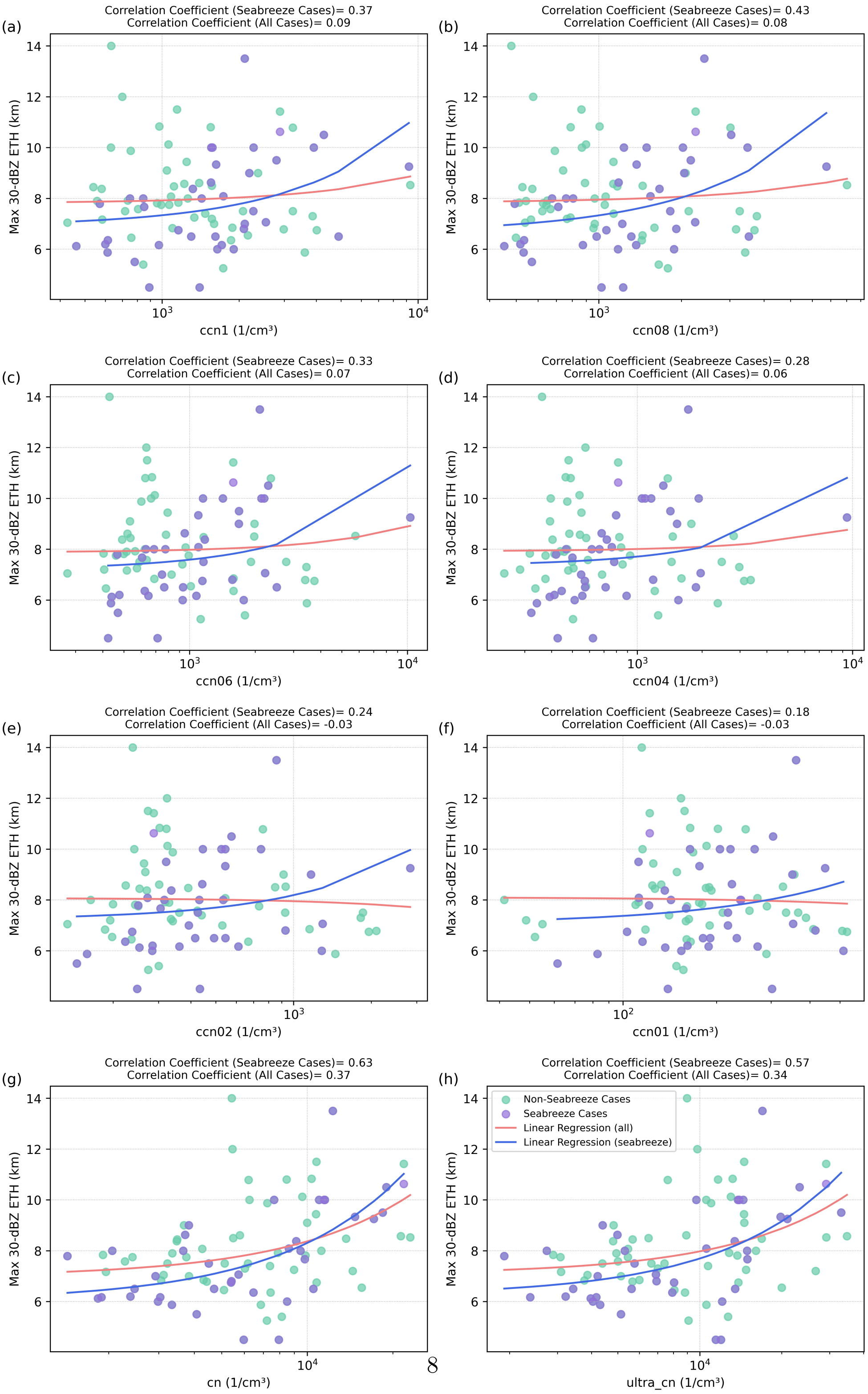

Fig. 5 Scatter plots showing aerosol number concentrations versus maximum 30-dBZ ETH.

Table 1 List of Acronyms and Definitions

Acronym	Definition
ANM	Additive Noise Model
AOS	Aerosol Observing System
ARM	Atmospheric Radiation Measurement
ARO	Aerosol Number Concentrations
CAM-UV	Causal Additive Model with Unobserved Variables
CAPE	Convective Available Potential Energy
CCN	Cloud Condensation Nuclei
CIN	Convection Inhibition
DAG	Directed Acyclic Graph
DCC	Deep Convective Cloud
DML	Double/Debiased Machine Learning
ELR	Environmental Lapse Rate
ETH	Echo Top Height
LCL	Lifting Condensation Level
LFC	Level of Free Convection
LNB	Level of Neutral Buoyancy
MIX	Mixed-layer parcel
ML	Machine Learning
MU	Most Unstable parcel
NaN	Not a Number
Nccn01	CCN Number Concentration at 0.1% supersaturation
Nccn02	CCN Number Concentration at 0.2% supersaturation
Nccn04	CCN Number Concentration at 0.4% supersaturation
Nccn06	CCN Number Concentration at 0.6% supersaturation
Nccn08	CCN Number Concentration at 0.8% supersaturation
Nccn1	CCN Number Concentration at 1% supersaturation
Ncn	Number Concentration of Aerosol Particles (10–3000 nm)
NOAA	National Oceanic and Atmospheric Administration
NOTEARS	Non-combinatorial Optimization via Trace Exponential and Augmented lagRangian for Structure learning
Nufp	Number Concentration of Aerosol Particles (3–3000 nm)
RH	Relative Humidity below 5 km
SFC	Surface parcel
SOM	Self-Organizing Map
TRACER	TRacking Aerosol Convection Interactions ExpeRiment
WSR	Low wind shear between the surface and 5 km

Table 2 Performed sensitivity tests when using causal discovery models. 576 tests in total.

Test	Parameter
Air parcel	MU, MIX, SFC
Outcome variable	30-dBZ ETH, 15-dBZ ETH
Mesoscale circulation	Sea Breeze, All Cases
Causal discovery models	CAM-UV, NOTEARS
Distance to ARM site	30 km, 40 km, 50 km
Exposure variables	N_{ccn01}, N_{ccn02}, N_{ccn04}, N_{ccn06}, N_{ccn08}, N_{ccn1}, N_{cn}, N_{ufp}

Table 3 Performed sensitivity tests when using causal inference model, DML. 1728 tests in total.

Test	Parameter
Air parcel	MU, MIX, SFC
Outcome variable	30-dBZ ETH, 15-dBZ ETH
Mesoscale circulation	Sea Breeze, All Cases
Distance to ARM site	30 km, 40 km, 50 km
DAG	CAM-UV, NOTEARS
Model in DML	Random Forest, Gradient Boost, Linear Regression
Exposure variables	N_{ccn01}, N_{ccn02}, N_{ccn04}, N_{ccn06}, N_{ccn08}, N_{ccn1}, N_{cn}, N_{ufp}
